# Family-based treatment for transition age youth: parental self-efficacy and caregiver accommodation

**DOI:** 10.1186/s40337-018-0196-0

**Published:** 2018-06-06

**Authors:** Gina Dimitropoulos, Ashley L. Landers, Victoria E. Freeman, Jason Novick, Olivia Cullen, Marla Engelberg, Cathleen Steinegger, Daniel Le Grange

**Affiliations:** 1 Faculty of Social Work, Matheson Centre for Mental Health Research, University of Calgary, 4212-2800 University Way N.W., Calgary, Alberta Canada; 2 Department of Human Development and Family Science, Virginia Polytechnic Institute and State University, Falls Church, VA USA; 3 University Health Network, Toronto General Hospital, Toronto, Ontario Canada; 4 Department of Sociology, Mount Royal University, Calgary, Alberta Canada; 5 Adolescent Eating Disorder Program, North York General Hospital, Toronto, Ontario Canada; 6 Division of Adolescent Medicine, The Hospital for Sick Children and the University of Toronto, Toronto, Ontario Canada; 7 Department of Psychiatry, University of California, San Francisco, CA USA; 8 Hotchkiss Brain Institute, University of Calgary, 4212-2800 University Way N.W., Calgary, Alberta Canada

**Keywords:** Family-based treatment, Family therapy, Anorexia nervosa, Eating disorders, Transition age youth, Caregivers

## Abstract

**Background:**

Family-Based Treatment (FBT) is the first line of care in paediatric treatment while adult programs focus on individualized models of care. Transition age youth (TAY) with Anorexia Nervosa (AN) are in a unique life stage and between systems of care. As such, they and their caregivers may benefit from specialized, developmentally tailored models of treatment.

**Methods:**

The primary purpose of this study was to assess if parental self-efficacy and caregiver accommodation changed in caregivers during the course of FBT-TAY for AN. The secondary aim was to determine if changes in parental self-efficacy and caregiver accommodation contributed to improvements in eating disorder behaviour and weight restoration in the transition age youth with AN. Twenty-six participants (ages 16–22) and 39 caregivers were recruited. Caregivers completed the Parents versus Anorexia Scale and Accommodation and Enabling Scale for Eating Disorders at baseline, end-of-treatment (EOT), and 3 months follow-up.

**Results:**

Unbalanced repeated measures designs for parental self-efficacy and caregiver accommodation towards illness behaviours were conducted using generalized estimation equations. Parental self-efficacy increased from baseline to EOT, although not significantly (*p =* .398). Parental self-efficacy significantly increased from baseline to 3 months post-treatment (*p* = .002). Caregiver accommodation towards the illness significantly decreased from baseline to EOT (*p* = 0.0001), but not from baseline to 3 months post-treatment (*p* = 1.000). Stepwise ordinary least squares regression estimates of eating disorder behaviour and weight restoration did not show that changes in parental-self efficacy and caregiver accommodation predict eating disorder behaviour or weight restoration at EOT or 3 months post-treatment.

**Conclusions:**

Our findings demonstrate, albeit preliminary at this stage, that FBT-TAY promotes positive increases in parental self-efficacy and assists caregivers in decreasing their accommodation to illness behaviours for transition age youth with AN. However, changes in the parental factors did not influence changes in eating and weight in the transition age youth.

## Plain English summary

Family-Based Treatment for Transition Age Youth were delivered to 26 participants with Anorexia Nervosa (ages 16–22) and their families. This study evaluated how family members responded to eating disorder (ED) behaviours throughout the course of this treatment. This study also evaluated how confident family members felt about their ability to help their loved one with ED behaviours throughout the course of treatment. The impact of changes in parental responses and confidence in helping with eating disorder symptoms and weight gain was examined. Throughout the course of treatment caregiver accommodation to eating disorder behaviour decreased and feelings of parental self-efficacy increased. However, changes in parental self-efficacy and accommodation did not predict changes in eating disorder behaviour and weight restoration in transition age youth with AN.

## Background

Anorexia Nervosa (AN) is a life threatening mental health condition [1] with severe consequences such as cardiac failure, osteoporosis [2], increased risk for suicide [3, 4] and significant comorbidities such as anxiety, depression and substance use disorders [5]. To prevent severe and enduring presentations of AN it is important that effective early interventions be provided to promote the best outcomes [6]. There is evidence for the use of family therapies (FT) with adolescents with AN [7], including Family-Based Treatment (FBT). However, there is limited evidence-based practice models for transition age youth between the ages of 18 to 24 [8–10]. Researching effective treatments for this age group is a necessary next step in the treatment of AN.

### Family-based treatment for adolescent anorexia nervosa

There are a variety of family therapy approaches developed for the treatment of AN such as Family Therapies for AN (FT-AN) and Multi-Family Therapy for AN (MFT-AN) [11]. The current study has focused on treatment using manualized FBT. At this time, FTs for AN are the most efficacious treatment for medically stable adolescents with AN [12, 13], and FT-AN is considered an appropriate treatment for this population [14]. The current study focuses on a manualized form of FT-AN: Family-Based Treatment (FBT). This treatment is guided by five fundamental principles: an agnostic view of the illness, externalization of the illness as something separate from the adolescent, emphasis on increasing parental empowerment, a focus on restoring healthy eating, and the therapist as a consultant to the family [15]. When compared to other treatments for AN, FBT has been found to reduce the need for hospital admissions during treatment [16], and has superior treatment outcomes on eating disorder symptomatology at end of treatment when compared to individual treatment and full remission at 6- and 12-month follow-up [17].

### Family-based treatments for transition age youth with anorexia nervosa

Althought studies of FBT indicate that it is an effective treatment [18] and that for participants ages 9 to 19 years, there are not significant differences in treatment outcomes based on age [19], FBT is not commonly used in specialized adult eating disorder programs (EDPs). This is possibly becausefamilies are not intrinsically present in adult ED treatment, whereas families often accompany youth to assessments and treatment in the pediatric system. However, there may be special considerations for transition age youth (ages 16 to approximately 25) that may necessitate unique family involvement, vital to the treatment of their illness in both pediatric and adult care.

Transition age youth experience many transitions that define their developmental stage, such as post-secondary education, increased fiscal responsibility, changing geographic location, and increased personal responsibility and autonomy [20]. For transition age youth with AN, these markers of young adulthood are present despite the limitations of their illness. Dimitropoulos et al., [21] found that eating disorder clinicians identified that many transition age youth express a desire for age appropriate support from their caregivers, including negotiating levels of involvement and support for their growing independence and confidence to overcome their ED. Due to a better understanding of these concerns for young adult development, experts in the field have begun exploring how to adapt FBT for transition age youth [8, 9].

An open trial of FBT for transition age youth (FBT-TAY) that encouraged negotiation between supportive caregivers and the independence of the youth has recently shown promising outcomes for individuals with AN [9]. FBT-TAY was designed for those between the ages of 16 to 25 and is an adaptation of FBT, which emphasises a collaboration between the transition age youth and his/her family, while maintaining their age-appropriate autonomy. Another pilot study of FBT for Young Adults (FBT-Y), where a similar collaborative approach to treatment was used, also showed promise with 59% of participants who completed treatment retaining weight restoration at 12 months post-treatment [8]. These adaptations of FBT are important for those ages 16 to 25 who are going through major life transitions [22], as well as moving from the pediatric to adult health care system [23].

### Caregivers’ impact on family-based treatment

Parental self-efficacy has been identified as a potential mechanism of change that promotes positive treatment outcome in FBT [24, 25]. Parental self-efficacy, as measured by the Parents versus Anorexia scale (PvA), is the ability for caregivers to be empowered in terms of supporting weight gain in their child [26]. Behavioural support around weight gain is required as in FBT parents are responsible for the re-nourishment of their child through consistent meal support as well as halting ED behaviours such as purging or excessive exercise. When parents achieve confidence in the re-nourishment process during the first four sessions of FBT, the adolescent is more likely to be weight restored at the end of treatment, as well as greater reductions in symptoms of depression and anxiety [26, 27]. Further, Robinson et al. [26] found that parents undergoing FBT with their adolescent had significant increases in parental self-efficacy throughout treatment which was predictive of reductions in ED psychopathology as well. Taken together, parental self-efficacy seems an important factor for positive treatment outcomes for adolescents in FBT. However, there were no studies that directly assessed caregiver self-efficacy in FBT for transition age youth with AN.

Caregivers may also engage in behaviours that inadvertently exacerbate ED symptoms [27, 28]. Eating disorders often lead families to ‘re-organize’ such that monitoring and managing symptoms become the primary concern. When this occurs, family functioning can decline and caregivers can feel helpless [28]. When caregivers inadvertantly accommodate the illness, they are engaging in maladaptive behaviours such as avoiding social situations, allowing meal restrictions, or enabling their loved one to continue a strenuous exercise regimen [29]. In a recent study by Stillar et al. [30], caregivers that experienced more fear and self-blame were also more likely to allow recovery-interfering behaviours in their loved one. It is important to note that the longer a loved one has had an ED, the more accommodating caregivers become to the illness’ symptoms as measured by the Accommodation and Enabling Scale of Eating Disorders (AESED) [29]. It is clear that accommodation and enabling behaviors seen in caregivers can negatively influence parental self-efficacy and thus negatively impact treatment outcomes. To date, there are no studies directly assessing caregiver accommodation and enabling behaviours in FBT for transition age youth with AN.

### Aims

The primary aim of the study was to determine if parental self-efficacy and caregiver accommodation changed in a course of FBT-TAY from baseline to the end-of-treatment (EOT), and 3 months post-treatment. The second aim of the study was to determine if changes in parental self-efficacy and caregiver accommodation influenced changes in eating disorder behaviour and weight restoration at EOT and 3 months post-treatment in transition age youth with AN.

## Method

### Data collection

An open trial of FBT-TAY was conducted across one adult and two pediatric hospital sites in Ontario, Canada between August 2014 and September 2016. The acceptability, feasibility, and impact of FBT-TAY on eating disorder behaviors and weight restoration is described by Dimitropoulos et al. [9]. Three hospital sites were selected to ensure a diverse sample of adolescents and young adults from pediatric and adult speciality ED clinics was obtained. Each hospital contained a specialized EDP and participants were recruited at the point of assessment for admittance into the EDP, as well as through community advertising. During initial assessment or a the time of self-referral from the community, confirmation of diagnosis was made by a psychologist or psychiatrist and if a potential participant was still interested in the study, they then met with the study coordinator and participated in informed consent. For an in depth description of the treatment please see Dimitropoulos et al., 2017 [9].

All potential participants were offered treatment as usual (TAU) or FBT-TAY. FBT was the TAU in both pediatric hospital sites (ages 12–18). In the pediatric hospitals, potential participants were offered either traditional FBT or FBT-TAY. At the adult hospital site, TAU was an intensive cognitive behavioral therapy (CBT) based group program within an inpatient (average stay of 5 months) or day hospital (average stay of 5 weeks) setting. In the adult hospital, family therapy was an optional component of treatment. At the point of assessment, if a potential participant met eligibility criteria (described below) for the FBT-TAY open trial, they were referred to the study coordinator. Potential participants were asked to identify at least one caregiver to participate in treatment. To support the autonomy of the transition age youth, caregiver selection was entirely left to their discretion and was defined broadly to include parents, guardians, siblings, partners, extended family and/or friends. All caregivers and participants with an eating disorder gave informed consent and were then assigned to a FBT-TAY study therapist. Further details about the methodology and participants are described by Dimitropolous et al. [9].

### Family-based treatment for transition age youth

**Fig. 1:**
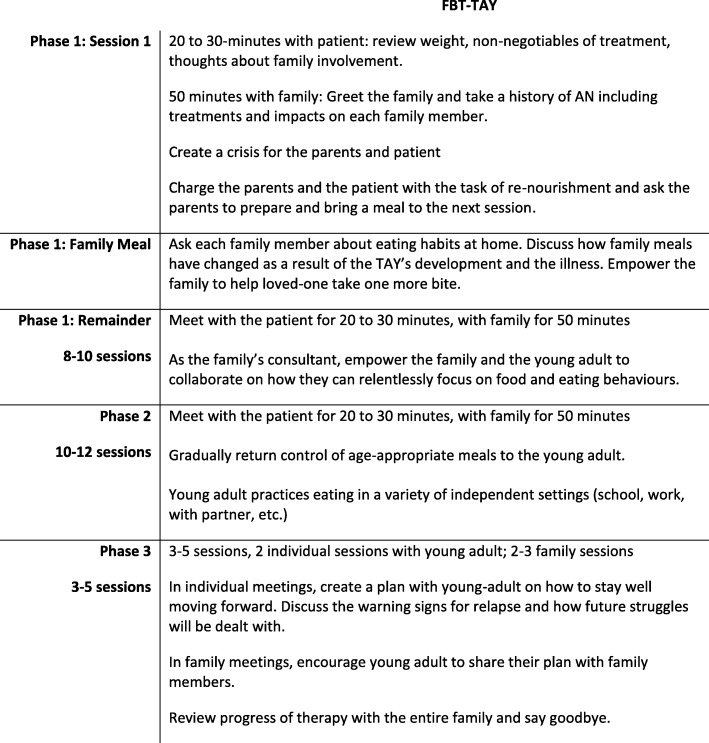
Summary of Family-Based Treatment for Transition Age Youth

FBT-TAY includes 25 sessions over the course of three phases [31]. These phases and their goals are briefly outlined in Fig. 1. Caregivers were integrally involved in each phase in the treatment. During phase one, the therapist worked collaboratively with the transition age youth to identify ways that their caregivers could specifically support them with meal support and symptom management. Explicitly, the caregivers were asked to support the transition age youth during mealtimes and monitor symptoms. The transition age youths were asked to be responsible for communicating to their families what type of support they required to assist with recovery from the eating disorder. In this way, the transition age youth had input in how their treatment was delivered. In phase two of treatment, the entire family was encouraged to allow the transition age youth more independent eating in a variety of normal situations (e.g., on a university campus, at work, with friends). Finally, in phase three, the transition age youth was asked to develop a plan for maintained recovery and shared this plan with their caregivers. The caregivers were tasked with being the first point of contact should a struggle with the eating disorder behaviours re-emerge.

#### Participant and caregiver characteristics

A total of 26 young people participated in this study (*M* = 18.15 years, *SD* = 2.11). The majority of participants were female (96.2%), Caucasian (61.5%), single (88.5%), living with family or relatives (92.3%), unemployed (53.9%), and had completed some high school education (46.2%). A total of 39 caregivers (23 mothers, 16 fathers) (*M* = 50.59 years old, *SD* = 6.80) were included in the analysis. Two parents did not complete the questionnaires required for inclusion in the analysis. The majority of caregivers were Caucasian (66.7%), married/partnered (79.5%), employed full-time (74.4%), and had an undergraduate university degree or higher (61.5%). See Table 1 for information regarding caregiver demographics.

**Table 1:** Caregiver Characteristics (*n* = 39)

	*n*	Percent or Mean	*SD*
Age		50.59	6.80
Gender			
Female	23	58.97	
Male	16	41.03	
Race			
Caucasian	26	66.67	
Non-Caucasian	13	33.33	
Marital Status			
Single	8	20.51	
Partnered/Married	31	79.49	
Living Situation			
With family, relatives, friends or partner	36	92.31	
Alone	3	7.69	
Education			
High school diploma	3	7.69	
Undergraduate university degree	24	61.54	
Some graduate education	1	2.56	
Graduate degree or higher	11	28.21	
Employment			
Part-time	5	12.82	
Unemployed	2	5.13	
Employed full-time	29	74.36	
Homemaker	2	5.13	
Disabled	1	2.56	
Religion			
Christian	21	53.85	
None	8	20.51	
Jewish	6	15.38	
Hindi	3	7.69	
Muslim	1	2.56	

## Measures

To assess the impacts of FBT-TAY on caregiver accommodation to the illness, parental self-efficacy, participant eating disorder behaviour, and weight restoration, analyses were performed across three timepoints: pre-treatment, post-treatment, and 3 months post-treatment.

### Instruments administered to transition age youth

#### Eating disorder behaviour

The Eating Disorder Examination Questionnaire (EDE-Q) is a 33-item, self-report measure which comprises four subscales (Restraint, Weight Concern, Shape Concern and Eating Concern) [32]. Each subscale has demonstrated excellent reliability and validity [33]. The EDE-Q global score is calculated by averaging all subscale scores.

#### Weight restoration

For each participant aged 16–20, the weight restoration goal was comprised their median BMI (kg/m^2^) according to age and sex [34]. For each participant aged 20.1–22, the weight restoration goal was a BMI of 20.0. At baseline, each participant’s height and weight was measured by the intake nurse. Subsequently, each participant’s weight was measured at the end of treatment and was self-reported for the EDE-Q three-month follow-up questionnaire. The BMI achieved by each participant aged 16–20 was divided by the median BMI for their age and sex to calculate weight restoration at baseline, end of treatment, and 3 months post-treatment. The BMI achieved by each participant aged 20.1–22 was divided by 20.0 to calculate weight restoration at each timepoint.

### Caregiver instruments

#### Parental self-efficacy

The Parents versus Anorexia (PvA) scale was developed to assess parental self-efficacy in the role of re-nourishing a child back to health [35]. This instrument has seven items rated on a five-point Likert Scale ranging from strongly disagree to strongly agree. Scores range from seven to 35. Higher scores indicate greater self-efficacy [35]. Parental self-efficacy was measured at baseline, end of treatment, and at 3 months post-treatment for mothers and fathers.

#### Caregiver accommodation

The Accommodation and Enabling Scale for Eating Disorders (AESED) is a 33-item self-report scale which is used to assess the degree to which caregivers accommodate and enable illness behaviours in their loved one [29]. Responses are measured on a five-point Likert Scale ranging from zero (never) to four (every day). The total scores range from zero (0) to 132 with higher scores indicating greater enabling and tolerating of ED behaviours. This scale is made up of five subscales which have Cronbach’s alpha values between .77 and .90. For the purpose of this study, we only used the total score of the AESED. Caregiver accommodation was measured at baseline, end of treatment, and 3 months post-treatment for mothers and fathers.

### Missing data

Missing data was addressed using multiple imputation [36] The process of multiple imputation involves multiple copies of a dataset being created and the missing values being replaced by imputed values. These imputed values are “sampled from a predictive distribution based on the observed data” [Pg. 2, 37]. This procedure takes into consideration the uncertainty associated with the prediction of missing values by including appropriate variability within the multiply imputed values [37]. The data of every caregiver who provided data at baseline was analyzed, regardless of their completion or withdrawal. Multiple imputation is superior to other approaches to address missing data, such as mean substitution or listwise deletion [38].

### Data analyses

Analyses were performed in IBM SPSS Statistics Version 24. The primary objective of this study was to investigate changes in parental self-efficacy and caregiver accommodation from baseline to end of treatment and from baseline to 3 months post-treatment. Due to the unbalanced and correlated nature of the data, unbalanced repeated measures designs for parental self-efficacy and caregiver accommodation were conducted using generalized estimation equations (GEEs) to determine the time effect while controlling for each participant. Separate analyses were undertaken for each of the predictors (the PvA total score and the AESED total score) due to concerns with multicollinearity. Bonferroni adjusted *p* values are reported for each GEE that was performed.

The second objective of this study was to investigate the impact of parental self-efficacy and caregiver accommodation to the illness on eating disorder behaviour and weight restoration in the transition age youth with AN. Ordinary Least Square (OLS) regression estimates of eating disorder behaviour and weight restoration were conducted at end of treatment and 3 months post-treatment. Separate regression analyses were undertaken for each of the predictors (changes in PvA and changes in AESED) due to concerns with multicollinearity. First, we examined whether the changes in parental self-efficacy from baseline to end of treatment predicted eating disorder behaviour and weight restoration at the end of treatment. Second, we examined whether the changes in parental self-efficacy from baseline to 3 months post-treatment predicted eating disorder behaviour and weight restoration at 3 months post-treatment. Third, we examined whether changes in caregiver accommodation from baseline to end of treatment predicted eating disorder behaviour and weight restoration at end of treatment. Finally, we examined whether changes in caregiver accommodation from baseline to 3 months post-treatment predicted t eating disorder behaviour and weight restoration at 3 months post-treatment. Z-scores were created for each variable in order to standardize measurement. This process is imperative when performing OLS regressions from multiply imputed data [39].

## Results

### Changes in parental self-efficacy

Table 2 presents the unbalanced repeated measures designs of parental self-efficacy by treatment time-point showing a statistically significant time effect (χ^2^ = 11.95, *p* = .003). Pairwise comparisons revealed that the total mean score of parental self-efficacy did not significantly increase from baseline (*M* = 18.91, *SE* = 0.62) to EOT (*M* = 19.82, *SE =* 0.69; *p* = .398), but did increase significantly increased from baseline (*M* = 18.91, *SE* = 0.62) to 3 months post-treatment (*M* = 21.59, *SE* = 0.50; *p* = .002).

**Table 2:** Parental Self-Efficacy and Accommodation

	Baseline		End of Treatment		Three Month Follow-Up	
	n	Mean (SE)	n	Mean (SE)	n	Mean (SE)
Parental Self-Efficacy						
PvA Total Score	39	18.91 (0.61)	39	19.82 (0.69)	39	21.59 (0.50)
Significance	.003					
Caregiver Accommodation						
AESED Total Score	37	46.31 (3.62)	37	37.75 (3.13)	37	45.16 (3.17)
Significance	0.0001					

*PvA* Parents versus Anorexia scale, *AESED* Accommodation and Enabling Scale

### Changes in caregiver accommodation

Table 2 also presents the unbalanced repeated measures designs of caregiver accommodation by treatment time-point showing a statistically significant time effect (χ^2^ = 37.45, *p* = .0001). Pairwise comparisons revealed that the total mean score of caregiver accommodation significantly decreased from baseline (*M* = 46.31, *SE* = 3.62) to EOT (*M* = 37.75, *SE* = 3.13; *p* = 0.0001), but not from baseline (*M* = 46.31, *SE* = 3.62) to 3 months post-treatment (*M* = 45.16, *SE =* 3.17; *p* = 1.000).

### Effects of parental self-efficacy on eating disorder behaviour and weight restoration

Table 3 presents the OLS regression estimates for the effects of changes in parental self-efficacy on participant eating disorder behaviour and weight restoration at EOT and 3 months post-treatment. Changes in parental self-efficacy from baseline to EOT, or from baseline to 3 months post-treatment did not significantly predict eating disorder behaviour or weight restoration in the transition age youth.

**Table 3:** OLS Regressions for the Effects of Parental Self-Efficacy on Patient Eating Behaviours and Weight Restoration (*n* = 39)

	EDE Globa Score	Weight Restoration
	*B*	*B*
Parental Self-Efficacy		
Change in PvA total score from time 1 to time 2	0.09	−0.33
Significance	0.591	0.223
Parental Self-Efficacy		
Change in PvA total score from time 1 to time 3	−0.09	0.01
Significance	0.735	0.985

*PvA* Parents versus Anorexia scale, *EDE Global* Eating Disorder Examination Global score

### Effects of caregiver accommodation to the illness on the transition age youth participant eating disorder behaviour and weight restoration

Table 4 presents the OLS regression estimates for the effects of changes in caregiver accommodation on eating disorder behaviour and weight restoration at EOT and 3 months post-treatment. Changes in caregiver accommodation from baseline to EOT, or at 3 months post-treatment, did not significantly predict eating disorder behaviour.

**Table 4:** OLS Regressions for the Effects of Parental Accommodation to Eating Disorders on Patient Eating Behaviours and Weight Restoration (*n* = 37)

	EDE Global Score	Weight Restoration
	*B*	*B*
Accommodation		
Change in AESED total score from time 1 to time 2	0.03	−0.12
Significance	0.865	0.582
Accommodation		
Change in AESED total score from time 1 to time 3	0.05	0.19
Significance	0.873	0.579

*AESED* Accommodation and Enabling Scale for Eating Disorders, *EDE Global* Eating Disorder Examination Global score

## Discussion

The current study of FBT for transition age youth with AN aimed to explore how parental self-efficacy and caregiver accommodation to the illness changed over the course of treatment. We further sought to identify whether such changes in parental self-efficacy and caregiver accommodation were predictive of changes in eating disorder behaviour and weight in the transition age youth at EOT and/or three-months post-treatment. We found that parental self-efficacy increased, but not significantly, from baseline to EOT. However, parental self-efficacy increased significantly from baseline to 3 months post-treatment. The results further revealed that accommodation to the illness decreased significantly from baseline to EOT, but not from baseline to 3 months post-treatment. Neither parental self-efficacy nor caregiver accommodation predicted change in ED symptoms and weight in the transition age youth at EOT or 3 months post-treatment. Overall, this study demonstrated that caregivers became increasingly more confident in their ability to support their loved one with AN, and perceived themselves as engaging less with/or permitting fewer ED symptoms throughout treatment. However, these changes did not predict change in the transition age youth’s eating disorder symptoms or weight.

### Change in parental self efficacy and Accomodation Behaviours

Transition age youth are between pediatric and adult systems of care, which requires special treatment attention to their unique developmental challenges. The experience of both wanting the support of family and friends while also negotiating boundaries for increased autonomy and independence can complicate how caregivers and transition age youth work together to diminish AN behaviours. Previous research on adolescent FBT found decreased parental self-efficacy and fear was linked with higher accommodation to the illness [30]. When families participate in FBT, caregiver mood and anxiety improve which correlates with increased self-efficacy [26].

It is important to note that the tool used to assess parental self-efficacy, the PvA, was developed for and is primarily used to assess empowerment in the context of child and adolescent treatment of EDs using FT [35]. Items on the PvA scale include: “I feel equipped with the specific practical strategies for the task of bringing about the complete recovery of my child in the home setting”, “while parents are important, children with anorexia will never get better until they receive some sort of individual therapy themselves”, and “It is more my responsibility than my child’s to bring him/her to a healthy weight” [35]. These items indicate a scale used to understand parental empowerment in the context of child and adolescent EDs treated with FTs where parents are an integral part of the support young people need as they are working to make behavioural changes [12, 13]. The items on the scale may not be applicable to transition age youth or a FBT-TAY model given the treatment goals: creating a collaborative approach between transition age youth and their families, individual therapy with the transition age youth, and individual development of recovery and maintenance plans in the final phase of treatment.

Interestingly, despite the limitations of the scale and the collaborative focus of FBT for TAY, caregivers still experienced a significant increase in parental self-efficacy from baseline to EOT. Working to empower parents and reduce fear and self-blame is a focus of FBT and other family therapies for AN (ex: FT-AN) [40]. Therefore, empowerment was prioritized in the development of FBT-TAY despite the increased autonomy in the transition age youth, which may explain the increased sense of self-efficacy throughout treatment and at 3 month follow-up. The majority of the transition age youth in the study sample were living at home (92.3%) and were struggling with AN-R or AN-BP. Within the FBTTAY model, parents were purposefully supported to feel empowered in helping their young adult with eating and recovery. The young adult was purposefully supported to develop acceptance of this parental support. This was achieved by the therapist acting as consultant to both the transition age youth and their caregivers. For example, therapists provided psychoeducation of the potential benefits of family involvement in meal support, particularly in the early phase of treatment; given the severity of complications that can develop when an individual is suffering with an ED [1–5], transition age youth may benefit from time limited support from their caregivers that would not typically be considered age appropriate.

The collaborative approach outlined above may be counter intuitive to families at this life stage where it is developmentally appropriate for young adults and parents to begin separating financially, geographically, and shifting their relationship from one of dependence to interdependence [20, 22]. However, FBT-TAY therapists collaborated with families to help them understand that short-term parental support may help transition age youth eventually achieve independent, age-appropriate, levels of autonomy around food and eating. FBT-TAY progressively focused on the development of healthy eating behaviours in a wide variety of settings such as at home, school and with persons outside the family. In phase 1 of treatment parents provided significant support for eating in a variety of situations while in phase 2, control of eating across various situations was gradually shifted entirely back to the transition age youth. This was focused upon so that transition age youth could practice and gain confidence eating and maintaining their recovery while also successfully engaging in the transitions inherent to emerging adulthood such as changes in school, work, finances and relationships [20, 22]. The use of FBT TAY is different from FBT for adolescents as young adults were asked to grant permission to their parents to take control of the provision of food preparation, serving and support during meals. It was also different from adult models of care where those over the age 18 are viewed as the most primarily responsible for their own recovery.

The result of increased parental self-efficacy over the course of FBT-TAY provides context for the caregiver’s significant decrease in accommodation behaviours over the course of treatment. Despite the unique features of transition age youth, caregivers in our sample were comparable to other adolescent and adult caregiver samples in terms of baseline accommodation and enabling scores [41–44]. The behavioural focus on FBT for transition age youth supports the identified caregivers, in every session, to collaborate with their transition age youth to support a reduction of eating disorder symptoms and facilitate weight gain. This treatment focus may explain why caregivers experienced a significant decrease in accommodation behaviours, however, the effects of this change were not maintained at 3 months post-treatment, indicating that this change may have been treatment dependent.

### Change in caregivers associated with change in transition age youth ED outcomes

In the current study, neither parental self-efficacy nor accommodation predicted change in eating disorder symptoms. This differs from past literature in the treatment of adolescent AN has found that parental self-efficacy increases during treatment with FBT [45]. In a study of 121 adolescents with AN, Byrne et al. (2015) found that families randomized to FBT had significantly greater increases in parental self-efficacy which was predictive of greater weight gain by EOT [24]. Most recently, parental self-efficacy has been found to be a significant mediator for increased weight gain in the adolescent by session 10 of FBT [46]. The transition age youth in the current sample differed from previously studied adolescents, and there was no predictive effect of change in parental self efficacy for FBT-TAY.

FBT-TAY differs from standard manualized FBT [15]. It begins with a very close collaboration with the transition age youth and parents and then an important goal of the therapy is to promote autonomous and independent eating in the transition age youth as well as age appropriate life re-integration (e.g..: return to College/University, living independelty and/or with peers or partner, etc.). This heightened focus on the transition age youth’s autonomy may account for why changes in caregiver’s self efficacy was not predictive of ED outcomes for the transition age youth. In the treatment of pediatric AN with standard FBT, it has been found that while parental self-efficacy is a significant predictor of weight gain, adolescent’s own self-efficacy is not [24] which again reinforces that the age appropriate nature of behavioural control by a caregiver may be different in adolescent vs transition age youth with AN.

Therapies that swiftly target caregiver beliefs about their ability to support a loved one are very important in facilitating their efforts to address disordered eating particularly during meal times. A caregiver’s ability to cope and provide support is impacted over time by feelings of fear and self-blame which erode a caregiver’s efficacy (e.g., accommodating to symptoms of restriction, purging, or over-exercise) [27, 28, 42, 47, 48]. The erosion of previously held caregiving skills can quickly spark a cyclical relationship between those with AN and their caregivers that makes the illness harder to overcome and increases the degree and duration of burden on caregivers [49]. Caregivers often experience social isolation, stigma, psychological distress [50, 51].

### Clinical implications and future directions

It is possible that FBT-TAY can be effective in halting or reversing caregiver disempowerment as well as decreasing accommodation behaviours in caregivers during the course of treatment. This is an important advancement in terms of our knowledge of the role of caregivers in the treatment of EDs in transition age youth. Currently, care for transition age youth receiving treatment in adult systems do not routinely involve caregivers, but instead prioritize individual or group-based models. Therefore, caregivers who are still intimately involved in the daily life of transition age youth are typically excluded from treatment of those over the age of 18. This is a problematic systemic issue that the current study proposes can be amended by introducing a developmentally appropriate model of treatment that recognizes the role that parents can play in supporting their adult child while also empowering the individual with the illness. Programs should begin to identify the unique needs of transition age youth and their caregivers to better serve this population. Future research should establish the efficacy of FBT-TAY via a randomized controlled trial. We also recommend furture research evaluating FBT-TAY to other family based treatment modalities like FT-AN or MFT-AN [11].

### Strengths and limitations

FBT-TAY was manualized and all study therapists were provided initial training followed by weekly supervision throughout the study. Even though the study used a novel model of treatment, it was developed using direct feedback from clinicians focused on working with transition age youth with AN [21]. Finally, the study sample and setting included both pediatric and adult programs which increases the real-world applicability of study findings to transition age youth accessing eating disorder treatment. The present study was not without limitations. We recruited a diverse range of caregivers in terms of age (range of 40–71 years), and gender (58.54% mothers), however, most were Caucasian (65.98%), held a college education or higher (90.25%), and were within an hour’s drive from a major city centre, limiting the generalizability of our findings. The study recruited a modest number of individuals with AN and their caregivers, and was only able to follow participants for 3 months post-treatment which is not sufficient to ascertain the long-term impacts of the FBT-TAY on caregivers.

## Conclusion

The current study is the first to describe the impact of FBT-TAY on caregivers of transition age youth with AN Acknowledging the unique needs of transition age youth who are between pediatric and adult systems of care is an important line of inquiry given the limited involvement of family members in treatment for older adolescents and young adults. The current study aimed to identify if involving family in the treatment of transition age youth using FBT-TAY would elicit benefits in the caregivers. This study demonstrates that FBT-TAY has promise and may lead to changes in caregivers of transition age youth with AN presenting for treatment in both pediatric and adult programs. Future studies should assess the impact of treatments on caregivers given the mental health impacts (e.g. depression, anxiety, burden) of caregiving (e.g. meal support, attending appointments, monitoring symptoms), and the positive treatment impacts for those with an ED (e.g. reduced hospital stays, decreased ED psychopathology, and increased quality of life).

## Abbreviations

AESED: Accomodation and enabling scale for eating disorders
AN: Anorexia nervosa
AN-BP: Anorexia nervosa binge purge sub-type
AN-R: Anorexia nervosa restrictive sub-type
CBT: Cognitive behavioral therapy
EDPs: Eating disorder programs
EDs: Eating disorders
EOT: End of treatment
FBT: Family-based treatment
FBT-TAY: Family-based treatment for transition age youth
FT: Family therapy
MFT: Multi-family therapy
PvA: Parents versus anorexia scale
RCT: Randomized controlled trial
TAU: Treatment as usual
TAY: Transition age youth
